# SARS-CoV-2 infection in the Indigenous Pataxó community of Southern Bahia, Brazil: second wave of transmission and vaccine effects

**DOI:** 10.1590/0102-311XEN112724

**Published:** 2025-04-25

**Authors:** Luciano Rodrigues Reis, Maria Helena Feres Saad

**Affiliations:** 1 Instituto Oswaldo Cruz, Fundação Oswaldo Cruz, Rio de Janeiro, Brasil.

**Keywords:** COVID-19, COVID-19 Vaccines, Indigenous People, SARS-CoV-2, COVID-19, Vacinas Contra COVID-19, Povos Indígenas, SARS-CoV-2, COVID-19, Vacunas Contra la COVID-19, Pueblos Indígenas, SARS-CoV-2

## Abstract

Indigenous people are at risk of several infectious diseases, including viruses that affect the respiratory system. In a previous study, we demonstrated how the Pataxó ethnic group, in the southernmost region of Bahia State, Brazil, was disproportionately affected during the first wave of COVID-19. Here, we provide an overview of how this community was affected by the second wave of the disease, evaluating the impact of vaccination on SARS-CoV-2 transmission. Prospective study data was grouped by Epidemiological Weeks 3/2021-43/2022, during which vaccine effects were analyzed and new variants of concern (VOC) emerged. The second wave produced a decreasing trimodal moving average curve, with an incidence rate of 4,407.2/100,000 inhabitants. Mobility and precarious work situations linked to tourism and craft trade increased infection rates in some villages. Risk factors for infection and severity (female sex, older age, and comorbidities) were determinants, but mortality was lower. Individuals with two doses of vaccine (Vac) developed more symptoms than the unvaccinated, but were less likely to have dyspnea. The mean time for COVID-19 symptoms to develop was longer in those with Vac (x̅ = 27 weeks) compared to those who received only one dose (x̅ = 12 weeks, p ≤ 0.001). Vac individuals who received booster shots, VacB1 and VacB2, had infection rates of 7.4% and 0%, respectively. The detrimental impact of COVID-19 once again highlights the persistence of health and socioeconomic inequities in this ethnic group. Moreover, the vaccines failed to prevent transmission, possibly due to mutated VOCs, but they may have protected this group against severe symptoms and extended the transmission period.

## Abstract (pt)

Os povos indígenas correm o risco de contrair várias doenças infecciosas, incluindo vírus respiratórios. Nosso estudo anterior demonstrou como a etnia Pataxó, na região mais ao sul do Estado da Bahia, Brasil, foi desproporcionalmente afetada na primeira onda da pandemia de COVID-19. Aqui, fornecemos uma visão geral da segunda onda de COVID-19 enfrentada por esta comunidade, avaliando como a vacinação impactou a transmissão do SARS-CoV-2. Os dados deste estudo prospectivo foram agrupados por Semanas Epidemiológicas (SE 3/2021-43/2022), durante as quais o efeito da vacina foi analisado e novas variantes de preocupação (VOC) surgiram. A segunda onda produziu uma curva de média móvel trimodal decrescente, com taxa de incidência igual a 4.407,2/100.000 habitantes. A mobilidade e as situações precárias de trabalho ligadas ao turismo e ao artesanato aumentaram a incidência em alguns povoados. Os fatores de risco para infecção e gravidade (sexo feminino, idade avançada e comorbidades) foram determinantes, mas a mortalidade foi menor. Indivíduos com duas doses de vacina (Vac) desenvolveram mais sintomas do que os não vacinados, mas eram menos propensos a ter dispneia. O tempo médio para o desenvolvimento dos sintomas de COVID-19 foi maior (x̅ = 27 semanas) naqueles sob Vac do que aqueles que receberam uma dose da vacina (x̅ = 12 semanas, p ≤ 0,001). Os Vac que receberam doses de reforço (VacB1 e VacB2) tiveram taxas de infecção de 7,4 e 0%, respectivamente. O impacto prejudicial do COVID-19 mais uma vez destaca a persistência das desigualdades socioeconômicas e de saúde neste grupo étnico. Além disso, as vacinas foram incapazes de prevenir a transmissão do vírus (possivelmente debido a VOCs mutantes), mas podem ter protegido indivíduos contra sintomas graves e prolongado o tempo de transmissão.

## Abstract (es)

Los pueblos indígenas corren el riesgo de contraer diversas enfermedades infecciosas, incluidos los virus respiratorios. Nuestro estudio anterior demostró cómo el grupo étnico Pataxó, en la región más meridional del estado de Bahía, Brasil, se vio afectado de manera desproporcionada en la primera ola de la pandemia de la COVID-19. En este estudio realizamos una descripción general de la segunda ola de la COVID-19 que enfrenta esta comunidad, con el fin de evaluar cómo la vacunación ha impactado la transmisión del SARS-CoV-2. Los datos de este estudio prospectivo se agruparon por Semanas Epidemiológicas (SE 3/2021-43/2022), durante las cuales se analizó el efecto de la vacuna y surgieron nuevas variantes preocupantes (VOC). La segunda onda generó una curva de media móvil trimodal decreciente, con una tasa de incidencia igual a 4.407,2/100.000 habitantes. La movilidad y las situaciones de precariedad laboral vinculadas al turismo y a la artesanía han aumentado la incidencia en algunos pueblos. Los factores de riesgo de infección y gravedad (género femenino, edad avanzada y comorbilidades) fueron determinantes, pero la mortalidad fue menor. Los sujetos con dos dosis de vacuna (Vac) desarrollaron más síntomas que los no vacunados, pero tenían menos probabilidades de presentar disnea. El tiempo medio hasta el desarrollo de los síntomas de la COVID-19 fue mayor (x̅ = 27 semanas) en aquellos con Vac que en aquellos que recibieron una dosis de la vacuna (x̅ = 12 semanas, p ≤ 0,001). Las personas con Vac que recibieron dosis de refuerzo (VacB1 y VacB2) tuvieron tasas de infección del 7,4 y 0%, respectivamente. El impacto perjudicial de la COVID-19 pone de manifiesto una vez más la persistencia de las desigualdades socioeconómicas y sanitarias en este grupo étnico. Además, las vacunas no pudieron prevenir la transmisión del virus (posiblemente con VOC mutantes), pero pueden haber protegido a los individuos contra síntomas graves y prolongado el tiempo de transmisión.

## Introduction

Coronavirus disease 2019 (COVID-19) causes severe acute respiratory infection and spread rapidly worldwide, impacting socially disadvantaged groups, including Indigenous populations in Brazil ^1^ ^,^ ^2^ ^,^ ^3^ ^,^ ^4^.

Brazilian Indigenous people are at risk of several infectious diseases, such as tuberculosis, intestinal parasitic infection, among others ^5^ ^,^ ^6^. This is probably due to the long-standing inequities that result in poor socioeconomic and sanitary conditions, along with malnutrition and other morbidities such diabetes, hypertension, heart diseases, etc. ^6^ ^,^ ^7^ ^,^ ^8^. Therefore, COVID-19 is another biological weapon threatening the health of Indigenous populations.

Despite Brazil having a healthcare system that aims to ensure primary care within Indigenous territories considering social, cultural, and geographical diversity, there are disparities in access to health services ^9^ ^,^ ^10^ and in COVID-19 data ^11^ ^,^ ^12^. Questions about the reliability of official data and the sufficient availability of diagnostic tests reflect an inaccurate assessment of the real impact of COVID-19 on morbidity and mortality in different ethnic groups ^11^ ^,^ ^12^. The potential for asymptomatic transmission further complicates this scenario, especially in regions lacking rapid detection mechanisms ^13^.

During the first year of the pandemic, these inequities and Indigenous communities’ lack of access to healthcare rapidly increased COVID-19 transmission and infection rates compared to the general population, with varying distribution across different regions of the country ^1^ ^,^ ^14^. However, a study carried out in Mato Grosso do Sul State revealed a higher number of cases and death among non-Indigenous populations compared to Indigenous ones, suggesting a positive impact of prioritizing vaccination for the latter ^15^.

Racial/ethnic and socioeconomic inequalities are also related to positive COVID-19 diagnoses, as Indigenous people with low socioeconomic status were twice as likely to test positive for COVID-19 compared to the general population ^16^. Additionally, mortality among Indigenous people was 16.7% higher than that observed in the general Brazilian population ^3^. Hospital mortality rates across all age groups were also higher among Indigenous people compared to other color/race categories ^2^ ^,^ ^17^. Factors such as marginalization, advanced age, and comorbidities further increase the risk of COVID-19 lethality among Indigenous individuals ^1^ ^,^ ^14^. These communities also tend to delay seeking care, which can lower their chances of survival in severe cases ^18^. The higher COVID-19 mortality rate among Indigenous populations in the first year of the pandemic was consistently observed in different regions of the country ^2^ ^,^ ^3^ ^,^ ^17^.

In a previous study ^19^, for the first time, the initial 490 days of COVID-19 transmission were analyzed on the Pataxó ethnic group in the southernmost region of Bahia State. Between May 22, 2020 and October 2, 2021, a total of 6,576 cases per 100,000 inhabitants confirmed the population’s vulnerability to infection. Moreover, cultural habits favored transmission, and the variation curve of cases at the end of the study period suggested a second wave of SARS-CoV-2 transmission in the Pataxó community. Although Indigenous populations in Brazil were prioritized by the COVID-19 vaccination program in Brazil, the progress of vaccination over time was diverse among different Indigenous Health Districts (DSEI, acronym in Portuguese) ^20^, with inadequate coverage for almost all strata of sex, region, socioeconomic index, and age ^21^. This can lead to significant disparities in access and health outcomes, especially for marginalized communities. Some authors have shown that, even with the reduction in COVID-19 incidence and mortality due to vaccination among Indigenous people, the cumulative incidence and mortality rates were higher than those observed in the general population across the country ^20^ ^,^ ^22^ ^,^ ^23^.

Although the understanding of the pandemic has significantly improved, its different impacts on the various ethnicities in the country remain unknown. Specific knowledge is important to better adapt public policies to the real needs of target populations. Therefore, we developed a prospective sectional study to continue to describe epidemiological data on the second wave of COVID-19 among the Pataxó community on the southernmost region of Bahia and evaluate the vaccination impact on SARS-CoV-2 transmission.

## Methods

A prospective cross-sectional study was carried out in Indigenous communities of the Pataxó ethnicity living in the municipalities of Porto Seguro and Santa Cruz Cabrália, in the southernmost region of Bahia State, which accounts for 90% of the total Pataxó population. Detailed description of the Pataxó community is available in our previous study ^19^. It is estimated that the community is composed of approximately 10,000 Indigenous people distributed in an area of ​​430.6109km^2^. They are organized in 24 villages, mainly in the municipalities of Porto Seguro and Santa Cruz Cabrália, occupying an average area of 770km^2^ that comprise seven Indigenous Lands: Aldeia Velha (≅ 1.465 inhabitants), Barra Velha (≅ 4.649 inhabitants), Coroa Vermelha, Coroa Vermelha Gleba C and Coroa Vermelha Ponta Grande (≅ 3.037 inhabitants), Imbiriba (≅ 721 inhabitants), and Mata Medonha (≅ 250 inhabitants) ^24^. In recent decades, this community has been the target of state and federal public actions, mainly related to land demarcation and other socioeconomic and sanitary issues ^19^.

The individuals were followed up during the study period (Epidemiological Weeks [EW] 37/2021-43/2022). Indigenous health teams and patient medical records provided for the accuracy of information of each reported case. Clinical-epidemiological data were retrieved from the Brazilian Ministry of Health databases. Other databases were also used: e-SUS Notifica (https://notifica.saude.gov.br) provided data of confirmed COVID-19 cases; the Brazilian National Immunization Program (SI-PNI, acronym in Portuguese; https://si-pni.saude.gov.br/) recorded vaccinations against COVID-19; and the Brazilian Mortality Information System (SIM, acronym in Portuguese; http://sim.saude.gov.br) and the Influenza Epidemiological Surveillance Information System (SIVEP-Gripe, acronym in Portuguese; https://sivepgripe.saude.gov.br) provided records of severe cases and death due to COVID-19.

**Figure 1:**
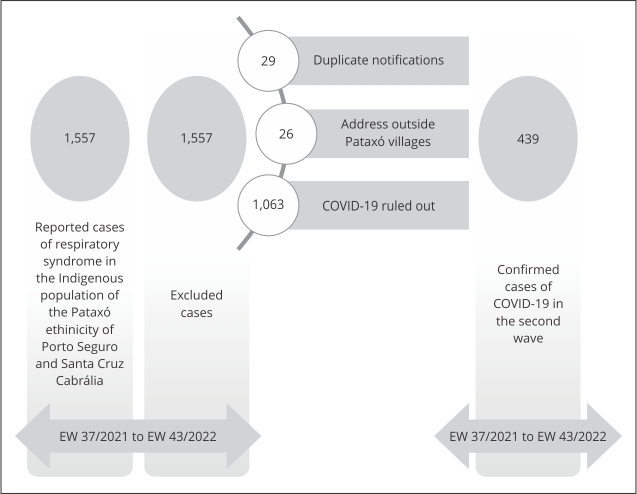
Flowchart of reported COVID-19 cases during its second wave in Indigenous Pataxó villages of Porto Seguro and Santa Cruz Cabrália, in the southernmost region of Bahia State, Brazil.

Note: no vaccination information available, duplicate records, atypical (heterologous) primary series, individuals with vaccination date outside the study period, and asymptomatic cases. EW: Epidemiological Week.

**Figure 2:**
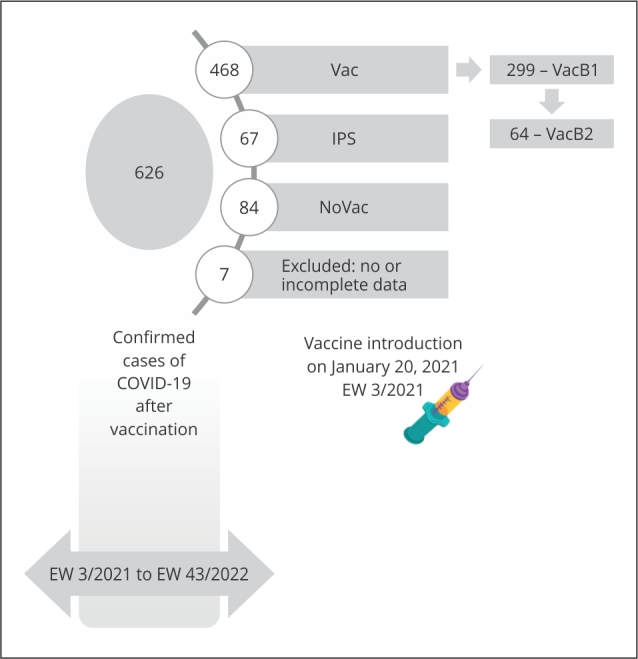
Flowchart of the vaccination status reported in Pataxó individuals throughout Epidemiological Week (EW) 3/2021-43/2022.

Note: NoVac (84/619, 13.6%): individuals without vaccination records. Vac (468/619, 75.6%), individuals with complete primary vaccination schedules (i.e., received two doses). IPS (67/619, 10.8%): individuals with incomplete primary vaccination schedules (i.e., received only one dose). VacB1 (299/619, 48.3%) individuals with completed primary vaccination schedules plus one booster dose. VacB2 (64/619, 10.3%), individuals with complete primary vaccination schedules and two booster doses.

COVID-19 cases were defined as those with laboratory confirmation or with final classification by clinical-epidemiological criteria. A flowchart of confirmed cases in the second wave is shown in Figure 1. Figure 2 shows data regarding vaccination against COVID-19. Those who received two homologous doses, of CoronaVac (Sinovac Life Sciences/Butantan) primary series, recombinant ChAdOx1 nCov-19 (AstraZeneca/Fiocruz), RNAm Comirnaty BNT162b2 (Pfizer-BioNTech), or single dose of recombinant Ad26.COV2.S (Johnson & Johnson/Janssen-Cilag), from EW 3/2021 to EW 43/2022, were considered immunized after 15 days, as were those immunized with the primary series plus a booster dose.

The number of weeks after vaccination did not show adherence to the normal curve (Kolmogorov-Smirnov’s test), so the comparison tests for this variable were Mann-Whitney’s test (two independent groups) or Kruskal-Wallis’s test (three or more independent groups) with post-hoc “pairwise” to identify significant differences. For vaccination coverage, we considered sample data extracted up to EW 9/2023. Age, sex, and municipality of residence were analyzed using Kruskal-Wallis’s test, while Mann-Whitney’s test was used to compare the number of weeks until COVID-19 symptoms were manifested. Statistical significance was set at p ≤ 0.05.

Incidence values and weekly moving averages of cases and deaths were calculated. The overall incidence and mortality rate were calculated based on data from the first ^10^ and second wave. The variables analyzed were village of residence, ethnicity, age, diagnostic criteria, symptoms, and comorbidities, as well as healthcare professionals who worked in Indigenous health teams. The overall spatial distribution of the incidence of the disease during the second wave, stratified by village, was determined using the QGIS software, version 3.20.3 (https://qgis.org/en/site/). The 2020 population estimates by village were based on the Brazilian Information System on Indigenous Health (SIASI, acronym in Portuguese) and referred only to Indigenous people assisted by the Brazilian Special Secretariat for Indigenous Health (SESAI, acronym in Portuguese).

This study was reviewed and approved by the Brazilian National Research Ethics Committee (CONEP, acronym in Portuguese; approval n. 34866720.1.0000.5248). All participants signed an informed consent form.

## Results

**Figure 3:**
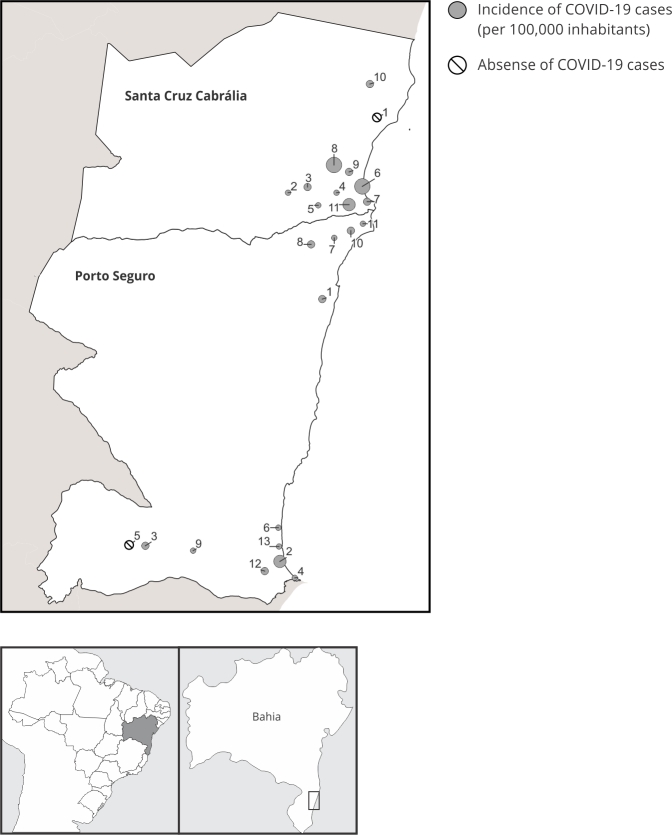
SARS-CoV-2 transmission distribution in the Pataxó villages located in the southernmost region of Bahia, Brazil, during the second COVID-19 wave.

The second wave of SARS-CoV-2 infection resulted in 439 cases in the Pataxó communities and an incidence rate of 4,407.2/100,000. The incidence rate considering the first and second wave was of 10,902.5/100,000. Notably, greater transmission occurred in Santa Cruz Cabrália (64.5%, n = 283, p < 0.01); however, the incidence was higher in some villages (Figure 3).

Villages: Porto Seguro - 1. Aldeia Velha; 2. Barra Velha; 3. Boca da Mata; 4. Bugigão; 5. Cassiana; 6. Imbiriba; 7. Jaqueira; 8. Juerana; 9. Meio da Mata; 10. Mirapé; 11. Ponta Grande; 12. Pará; 13. Xandó. Santa Cruz Cabrália - 1. Araticum; 2. Aroeira; 3. Agricultura; 4. Itapororoca; 5. Txihi Kamayurá; 6. Cruzeiro; 7. Coroa Vermelha; 8. Carajá; 9. Campo; 10. Mata Medonha; 11. Nova Coroa.

**Figure 4:**
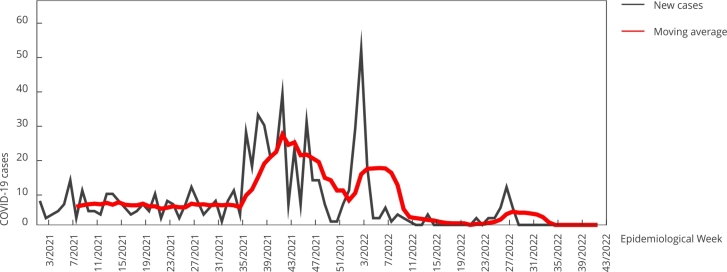
Number of new COVID-19 cases in the second wave per Epidemiological Week (EW 37/2021-43/2022) of notification, in the Pataxó ethnic group. Municipalities of Porto Seguro and Santa Cruz Cabrália in the southernmost region of Bahia, Brazil.

The weekly moving average curve of new cases during the second wave showed a decreasing trimodal peak pattern (EW 43/2021, EW 4/2022, and EW 28/2022) interspaced by low transmission levels (Figure 4). Sociodemographic characteristics revealed that the most affected were the Pataxó (97.7%), followed by healthcare workers (2.3%), females (60.8%), and adults of working age (19-60 years, 70%, x̅ = 36.5, σ = 10.9; p ≥ 0.09). Only 9.1% of all cases involved children (< 12 years, 40/439), and there was no significant difference between municipalities (p = 0.09).

Clinical-epidemiological or imaging criteria were used in only 1.4% (6/439) of reported cases. Despite the difficulties concerning inputs and diagnostic tests, 98.6% (433/439) of COVID-19 cases were diagnosed by laboratory criteria, with real-time quantitative polymerase chain reaction being the main one (59.2%, 260/439). Mobility outside the village of origin was present (13.2%, 58/439), with higher rates observed in the villages Barra Velha (Porto Seguro, 37.9%, 22/58) and Coroa Vermelha (Santa Cruz Cabrália, 17.2%, 10/58; p < 0.01). Regarding clinical profiles, there was a significant increase in the number of symptoms in the gastrointestinal (e.g., nausea, vomiting) and upper respiratory tract (e.g., runny nose, olfactory disturbance, sore throat, cough), as well as systemic symptoms (e.g., arthralgia, headache, fever) accompanied by a lower number of respiratory tract symptoms (e.g., dyspnea), observed in the Santa Cruz Cabrália villages from 2022 to 2021 (p ≤ 0.05). The year with the highest number of symptoms was 2022, although this was also the year with the lowest number of reported cases (Table 1).

**Table 1:** Median symptoms among Indigenous patients of the Pataxó ethnicity infected with COVID-19 during the second wave of its outbreak, stratified by year and municipality of origin.

Municipality/Year	n	Minimum	Maximum	Median	IQR	X̅	σ
Overall (n = 439)							
2021 *	486	1	11	4.0	2.0	4.0	1.9
2022 * **	156	1	11	4.0	3.0	4.6	2.2
Porto Seguro (n = 153)							
2021	215	1	9	3.0	2.0	3.7	1.7
2022	54	1	9	4.0	2.0	3.8	1.6
Santa Cruz Cabrália (n = 286)							
2021 *	271	1	11	4.0	3.0	4.2	2.0
2022 * **	102	1	11	4.5	3.0	5.0	2.3

X̅: arithmetic mean; σ: standard deviation; IQR: interquartile range.

Source: prepared by the authors, based on data from the e-SUS Notifica, 2022 (https://notifica.saude.gov.br).

* p ≤ 0.05;

** p ≤ 0.01.

Chronic heart diseases were common, including arterial hypertension (11.8%, 52/439) and diabetes (5.5%, 24/439). Deaths due to COVID-19 mostly occurred in older people (≥ 60 years, 60%, 3/5), followed by those with comorbidities (80%, 4/5) and low mortality rate (1.1%, 50.2/100,000), resulting in an overall indicator of 1.2% (130.5/100,000). The COVID-19 mortality rate between the sexes did not differ (p ≥ 0.05).

Vaccination began on January 20, 2021 (EW 3/2021). The transmission decreased to a baseline level, with few cases occurring during the first weeks of vaccine rollout, remaining consistent until EW 36/2021 (Figure 4). The mean number of cases over these 33 weeks was 180, and vaccination did not seem to change the dynamics during this period. However, when the second wave occurred, the cumulative number of new cases remained high in 2021 (n = 282), but there was a reduction of 44.3% (125/282) in 2022 (up to EW 43/2022). Despite initial resistance, adherence to the vaccination program soon increased, with 86.4% (535/619) of patients diagnosed with COVID-19 receiving at least one dose of the vaccine and 75.6% (468/619) receiving two doses. Therefore, on EW 9/2023, NoVac (individuals with no vaccination record) was 13.6% (84/619) (Figures 1 and 2), of whom 48.8% (41/84) were either below or above working age (33.3% < 10 years and 15.5% > 60 years).

Of those immunized with CoronaVac (67.8%, 420/619), most (93.1%, 391/420) received the Vac scheme (complete primary vaccination schedule), and 65% (273/420) received VacB1 (complete primary vaccination schedule plus one booster dose). Of the remaining, 15.2% (94/619) received BNT162b2 under Vac (65.9%, 62/94) and VacB1 (20.2%, 19/94) schemes, 3.2% (20/619) received ChAdOx1 nCov-19 under Vac (70%, 14/20) and VacB1 (35%, 7/20), and a single person (0.1%) received Ad26.COV2.S. The municipalities of residence did not differ in vaccination schedules (p > 0.05), and the vaccination coverage profile did not differ between sexes (p = 0.303). The overall number of individuals who were immunized with VacB1 was 48.3% (n = 299/619), and approximately half received ChAdOx1 nCov-19 as a booster (53.8%, 161/299), followed by BNT162b2 (33.4%, 100/299), Ad26.COV2.S (11.4%, 34/299), and CoronaVac (1.3%, 4/299). Overall, 10.3% (n = 64/619) of the population was immunized under the VacB2 schedule (complete primary vaccination schedule and two booster doses), of which Ad26.COV2.S (35.9%, 23/64), ChAdOx1 nCov-19 (34.4%, 22/64), and Pfizer (28.1%, 18/64) were the most frequently used booster.

**Figure 5:**
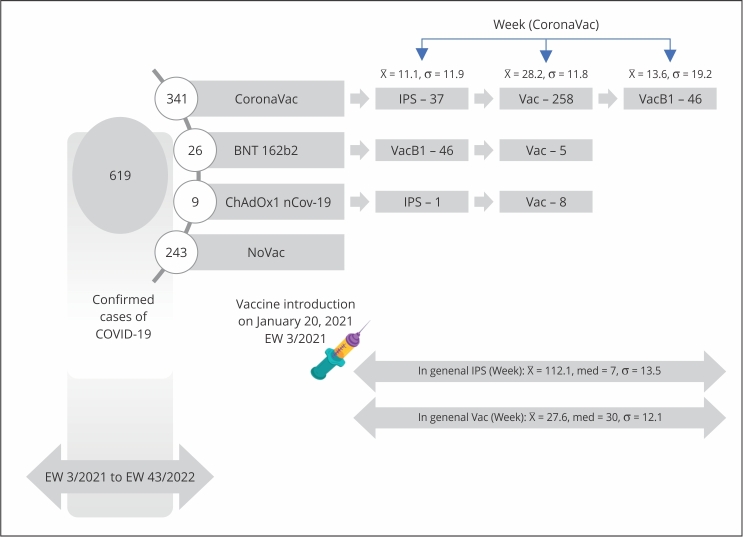
Status of vaccine and COVID-19 distribution in the Pataxó group from Epidemiologicval Week (EW) 3/2021 to 43/2022, in the municipalities of Porto Seguro and Santa Cruz Cabrália, southernmost region of Bahia, Brazil.

IPS: incomplete primary schedule; NoVac: no vaccination record; Vac: completed primary vaccination schedule; VacB1: Vac plus one booster dose. Vaccines: CoronaVac (Sinovac/Butantan); ChAdOx1nCov-19 (AstraZeneca/Oxford/Fiocruz); BNT162b2 (Pfizer/BioNTech); Ad26.Cov2 (Janssen/Johnson & Johnson).

Our analysis of COVID-19 cases according to vaccination status revealed that 39.3% were infected before receiving a vaccine dose (NoVac), with no significant differences between municipalities (p > 0.05). Higher transmission frequencies occurred among Vac individuals (43.7%), and, combined with those who had an incomplete primary vaccination schedule (IPS), they accounted for half of infections (53.2%); however, most were from Santa Cruz Cabrália (64.2%, p < 0.01). Notably, after recovering from the disease, 65.4% of NoVac individuals joined the vaccination program at EW 9/2023 (Figure 5). Of those under the VacB1 schedule, 7.4% were later infected with SARS-CoV-2, more frequently in Santa Cruz Cabrália than in Porto Seguro (60.9%, 28/46 versus 39.1%, 18/46; p ≤ 0.05). All patients were vaccinated using CoronaVac (Figure 5). None of those who received VacB2 were infected. Vac individuals showed a greater number of symptoms compared to NoVac (p ≤ 0.01). The number of symptoms remained unchanged in those who received booster shots (p = 0.838).

Figure 5 shows that, for those under the Vac scheme, the mean time for COVID-19 symptoms to develop was longer (x̅ = 27 weeks) compared to those with an IPS (x̅ = 12 weeks, p ≤ 0.001). Two doses of CoronaVac conferred protection for an average of 28 weeks (p ≤ 0,001), whereas a booster extended this period (x̅ = 13.6 weeks), which was longer regarding IPS (x̅ = 11.1, p ≤ 0,001). None of the Vac scheme participants developed COVID-19 symptoms prior to vaccination. Outcome deaths occurred in one individual with VacB1 (CoronaVac/Ad26. Cov2) and two with Vac (CoronaVac); they were older or had comorbidities considered risks for severe COVID-19 (60%, 3/5).

## Discussion

The COVID-19 pandemic sociodemographic profiles in the Pataxó community mirrored the overall Bahia population ^25^ and other Indigenous groups in Brazil ^26^. These data suggest a greater relevance of social, cultural, and demographic factors than that of biological factors regarding exposure to the virus and susceptibility to serious COVID-19 outcomes ^12^ ^,^ ^25^ ^,^ ^26^ ^,^ ^27^ ^,^ ^28^. Consistent with other ethnicities and non-Indigenous populations in Brazil, Pataxó children were less affected and generally had fewer COVID-19 symptoms ^20^ ^,^ ^26^ ^,^ ^29^. This may be due to several factors: lower prevalence of comorbidities ^20^ ^,^ ^30^; cross-immunity from previous infections with other coronaviruses and vaccines ^31^ ^,^ ^32^; higher proportion of lymphocytes ^33^; and more diverse colonization of microorganisms in the upper and lower respiratory tract as well as in the gastrointestinal microbiota. These microbial interactions and competition can limit the multiplication of SARS-CoV-2 ^34^ ^,^ ^35^. Migration is cultural among the Pataxó people; however, during the first wave ^19^, a lack of knowledge about the disease might have been a significant risk factor, while in the second wave, it was the precarious regular economic activities and resumption of work to avoid starvation. Therefore, it is not surprising that two-thirds of cases were reported in Santa Cruz Cabrália, where economic, tourism, and craft-related activities are concentrated (Figure 3).

Regarding clinical profiles, changes were observed in the number of symptoms, mainly in Santa Cruz Cabrália in 2022 and possibly because of the predominance of the Omicron variant in the region during this period. Omicron infection is less involved with the lower respiratory tract and has a lower probability of hospitalization ^36^ ^,^ ^37^ ^,^ ^38^. Moreover, the viral loads of Omicron subvariants, which are significant factors associated with severe disease outcomes, are generally lower in the lungs than in the nasal mucosa ^39^ ^,^ ^40^. Although determinants of severity are multifactorial, further studies are warranted to understand these aspects considering the emergence of new variants of concern (VOCs) and vaccination of Indigenous communities of Brazil.

During the second wave, a notable reduction in the incidence rate was observed compared to the first wave ^19^. However, the cumulative disease incidence (10,902.5/100,000 inhabitants), despite being lower than the national average (16,445.4/100,000) ^29^ and in the Bahia State (11,371.10/100,000) ^25^ during the same period, highlights the severity of transmission in this community. The first peak of the trimodal decreasing curve of the second wave (43/2021) coincided with the presence of the Delta variant, which was first identified in May 2021 ^41^ ^,^ ^42^. The Omicron variant was first identified in November 2021 and soon spread worldwide ^42^ ^,^ ^43^, including to Brazil ^42^ ^,^ ^44^. Nationally, the Omicron subvariant (BA.1) that emerged in the city of São Paulo was responsible for the COVID-19 outbreak from December 2021 to March 2022 ^42^ ^,^ ^45^. Although our study was not designed to identify viral variants transmitted in the Pataxó community, we can infer this variant was responsible for the second peak (EW 4/2022), since it coincided with its presence in the country. The same applies to the third peak (EW 28/2022), which might have been influenced by the emergence of additional Omicron subvariants (BA.4/BA.5), responsible for the new increase in disease cases predominantly from July 2022 to January 2023 ^42^. However, the increase in the number of symptomatic infections did not match the number of hospitalizations and deaths in regions with significant vaccine coverage ^29^ ^,^ ^46^ ^,^ ^47^. In Pataxó communities, it is likely that the vaccine coverage, even if it were not able to block disease transmission, provided a longer period of protection (33 weeks: EW 3/2021 to EW 36/2021). This, combined with immunity from natural infections and the lower virulence of circulating variants, might have contributed to the reduction in disease incidence in most villages, as well as to the decreasing trend observed in the rolling average of cases. This phenomenon has also been observed in other Indigenous communities in countries with high vaccination coverage ^20^ ^,^ ^26^.

The intrinsic functional abilities acquired by VOCs generally confer greater transmissibility, viral fitness, and immune evasion, causing significant reductions in the immune response by memory B cells and neutralizing antibodies, induced by natural infection with SARS-CoV-2 and vaccines based on the spike glycoprotein (or sometimes just the receptor-binding domain [RBD] or inactivated virus) of the wild-type Wuhan-Hu-1 strain ^48^ ^,^ ^49^ ^,^ ^50^ ^,^ ^51^ ^,^ ^52^ ^,^ ^53^ ^,^ ^54^ ^,^ ^55^ ^,^ ^56^ ^,^ ^57^ ^,^ ^58^. Our findings corroborate this, as almost half of the cases reported, after the introduction of the vaccination program, occurred among those who were vaccinated, but most of them progressed to a mild flu-like illness (98.8%). Additionally, 24% of the Pataxó community was NoVac or IPS, and, along with mutation events in the virus conferring greater immune escape from vaccine-derived immunity, this might have influenced the increased incidence in some villages and the consequent maintenance of the disease transmission chain. Even during the period of stability, SARS-CoV-2 was present in most villages. This stability was maintained for 33 weeks after the vaccination program. It is not possible to certainly determine if this extended the stability until the second wave, but it may be plausible, as the number of weeks of vaccination coverage until the emergence of disease symptoms was an average of 27 weeks.

In Brazilian Indigenous communities, as in the general population, varying schedules have been used for many individuals regarding the third dose of the vaccine (vaccine availability). This approach is in line with evidence suggesting that broader and more durable humoral responses were achieved using different third doses compared to three identical doses ^51^ ^,^ ^53^ ^,^ ^55^ ^,^ ^56^ ^,^ ^57^. In our study, after receiving a booster dose of the VacB1 series, protection remained for an average of nearly 14 weeks until the onset of symptoms, and no cases of VacB2 infection were recorded during the follow-up period. However, a nationwide study ^51^ found that the Anti-Spike IgG response after the first booster was greater than that after the second booster, suggesting that sequential booster doses may produce less pronounced responses.

Notably, NoVac adhered to the vaccination schedule (65.4%) following recovery from symptomatic COVID-19 infection. Studies have suggested that hybrid immunity (a combination of virally induced and vaccine-derived immunity) increases the neutralizing activity and affinity maturation of cross-reactive antibodies against SARS-CoV-2 variants after the second or third dose compared to the level of immunity in individuals who were never infected or those who only possess vaccine-induced immunity ^50^ ^,^ ^51^ ^,^ ^57^ ^,^ ^58^. Nevertheless, deaths occurred in older people and those with comorbidities that conferred a higher risk of developing severe COVID-19 outcomes. Moreover, the cumulative of the first and second disease-related mortality rate of 1.2% (140.5/100,000), although lower than the national average (1.9%; 324.9/100,000) ^29^ and the Bahia State (1.8%; 211.7/100,000) ^25^, was higher than the overall Indigenous mortality rate in the country (110.1/100,000) over the same period ^26^, suggesting the Pataxó community are more vulnerable to severe COVID-19.

Unlike other studies that described a more favorable disease course in women, we found sex-biased transmission, but not for severe disease or death ^13^ ^,^ ^28^ ^,^ ^59^. Additionally, Santa Cruz Cabrália had higher mortality rates, which may be explained by its proximity to urban areas (84.8%) with higher levels of tourism, whereas Porto Seguro villages, although more predominant in rural areas (67%) have better networks of healthcare services and therapies. Comorbidities were risk factors for severe disease outcomes but were more common in Porto Seguro. These data reinforce the importance of mitigation strategies and disease surveillance targeting older people and clinically vulnerable populations, along with actions that consider their ethnic, cultural, and sociodemographic characteristics.

## Conclusion

Pataxó communities have been heavily impacted by the COVID-19 pandemic. Cultural behavior and poor working conditions contributed to the spread of the virus. While vaccines and booster doses did not prevent transmission, they may have delayed it, which might have provided better protection against severe symptoms. However, the emergence of new VOCs could have influenced this outcome. To overcome vulnerability, high-quality holistic education, along with sustainable economic exploitation of their fertile lands for the benefit of the community, is urgent.
